# Supplementary material for: Acceptance and compliance with micronutrient powder (MNP) among children aged 6–23 months in northern Nigeria
Source: PLOS Glob Public Health. 2022 Oct 17;2(10):e0000961. doi: 10.1371/journal.pgph.0000961 (PMC10022258; doi:10.1371/journal.pgph.0000961)
Supplement: S1 Dataset — (ZIP) [file pgph.0000961.s008.zip › S8_Dataset/Direct Observations.zip!/Direct Observations/270615_K_DW_SU_1_DO.pdf]

1.1.1 Instrument F –Direct Observation Form

<<Instrument F Direct Observation Form>>

PI Name: PragyaMathema,Stephen Kodish
Study Title: Formative research to develop an infant and young child feeding (IYCF) intervention with micronutrient powder (MNP) for children aged 6 – 23 months
PI Version/Date: Version1.0/January 24, 2015

Date: 27/06/2015 Location: Rafin Gora

Data Collector: Samira Usman

Main goals of this direct observation are to answer the following questions:

1. In this household, what would be the best way(s) to give nutritional supplements to young children? Why?

2. In this household, describe the food sharing that you observe throughout the meal. Are there any predictable patterns?

3. In this household, who is favored and how is this favoritism expressed during meal preparation and eating?

Household Composition Table (Fill out as you do data collection)

INITIALS	SEX (M/F)	AGE (estimated)	Relationship to household	Description
MM	F	28yrs	Mother	Average height, blue yellow wrapper (Ankara) and blue short with black and white strike head tie and hijab
BB	F	17months	Daughter	Dark, wearing pink sleeveless and short
FF	M	36yrs	Father	Tall dark wearing brown trousers, top and cap
BB2	M	1yr	son	Dark wearing black trousers and green top
GM	F	50yrs	Grand mother	Tall dark wearing wrapper and head tie (Ankara)
TB	M	12yrs	Mothers brother	Tall, dark boy wearing a flowery shirt and black jeans short
NB	F	29yrs	Neighbor	Average height wearing yellow and blue wrapper, red and black top with black head tie
SB	M	3.5 yrs	Neighbor son	Wearing black and white short and black

				jeans

TIME	ACTOR INITIALS	RECIPIENT INITIALS	WHAT HAPPENED	FOOD	Reactivity
7:40	MM	BB	Mother fetch pap in a bowl and keep it in front of the baby, she starts drinking	Cold papa	
7:45	BB		Baby drinking the pap	Cold pap	
7:50	BB2		The baby's brother come to the house		
7:55	BB	BB2	The baby and her brother are playing		
8:00	FF		Father enter into the house and enter the room		
8:05	MM		Mother is sweeping her room and compound		
8:10	BB		Baby is easing herself on the ground		
8:15	FF,MM		Father went out and the mother is washing clothes		
8:20	MM		Mother washing the clothes		
8:25	MM	BB,BB2	Mother brings tuwon dawa with vegetable soup for the children and they started eating	Tuwon dawa and spinach leaves	

TIME	ACTOR INITIALS	RECIPIENT INITIALS	WHAT HAPPENED	FOOD	Reactivity
8:30	BB,BB2		The children are eating the food	Tuwon dawa with spinach leaves	
8:35	BB2	BB	The older brother is drinking water and he gives to the boy		
8:40	BB,BB2		The boy and her brother are playing with the sand		
8:45	MM		Mother enters the toilet		
8:50	BB,BB2		The baby and her brother are still playing with some other children that came into the house		
8:55	MM		Mother is washing her legs and face		
9:00	MM		Mother is going to grandmother house with the children		
9:05	MM		Still going to the grandmother house		
9:10	GM		Grandmother is washing plates, the mother greeted her and sat down		
9:15	MM		Mother left the house leaving the children in grandmother house		
9:20	BB2	BB	The brother is drinking water and give to the sister		
9:25	BB	BB2	Baby playing with her brother with sand outside the house		
9:30	BB,BB2		They are still playing		
9:35	BB		Baby playing with the other children in the community		
9:40	FF		Father comes to the grandmother house, we are sitting under the tree		
9:45	FF	BB	Baby is sitting on her father’s lap		
9:50	BB,BB2		Baby playing with her brother		
9:55	BB,BB2		Baby and her brother with some other neighbou children of the same age are playing outside the house under the tree with		

TIME	ACTOR INITIALS	RECIPIENT INITIALS	WHAT HAPPENED	FOOD	Reactivity
			grandmother sitting		
10:00	BB,BB2		They are still playing		
10:05	GM	TB	Grandmother sent the mother brother to call the mother (they are talking in language) I asked the grandmother she said she sent for her because she is going to the farm		
10:10	GM		Grandmother left for farm leaving the children with the neighbor under the tree		
10:15	BB,BB2		The children are playing		
10:20	BB,BB2		The children are still playing		
10:25	BB		Baby playing with the neighbor daughter		
10:30<br><br><br><br>10:35	MM		Mother just surfaces with bunch of leaves on her head, I greeted and introduce myself and she said no problem. I asked her what is this, she said tamarind leaves they use it for cooking tuwo, they called it tuwon tsamiya (tamarind) and the baby is sitting on her lap	Tamarind leaves	
10:40	MM	BB,BB2	Mother is removing catarrh from the son and the baby is playing on her lap		
10:45	MM.BB BB2		Mother sais we should go the house, she took the bunch of leaves placed on her head, took the baby and hold the son hand		
10:50	MM,BB BB2		On the way to the house		
10:55	MM,BB BB2		The mother and her children are sitting on the mat		
11:00	BB,BB2		The baby and her brother are playing		
11:05	BB		The baby is playing with MNP sachet		

TIME	ACTOR INITIALS	RECIPIENT INITIALS	WHAT HAPPENED	FOOD	Reactivity
11:10	BB,TB		The bay is still playing with the used sachet putting it in her mouth and using it as her phone saying hello The mother's brother comes into the house		
11:15	BB		The baby is touching my hijab (veil) and my bag	She is touching me because I am in their house	
11:20	BB,BB2		The baby is playing with her mother		
11:25	TB	BB,	The mother's brother goes to the room fetch tuwon dawa and spinach soup prepared with tamarind leaves, water. Pepper,s alt and magi. I asked the mother and she told me how the prepared it. The baby is feeding herself	Tuwon dawa with spinach and tamarind leaves	
		BB	The son took the tuwon dawa in a bowl add water in it and sugar mixed it starts drinking it		
11:30	MM	BB	Mother is backing the baby		
11:35	BB		Baby is playing with the remaining food in the bowl		
	MM	BB	Mother sent the son to buy razor blade		
11:40	MM	BB	Mother is cutting the baby's nails with the razor blade and equally removing the son's nails		
11:45	MM	BB,BB2	Baby lying on her mother laps and the mother is cutting her son nails while chatting with her neighbor		
11:50	BB,BB2		Mother cutting her nails, chatting with neighbor		
11:55			and the baby is playing and her brother is sleeping on a mat		

TIME	ACTOR INITIALS	RECIPIENT INITIALS	WHAT HAPPENED	FOOD	Reactivity
12:05	BB		Baby still playing		
12:10	MM	BB	Baby start making trouble and the mother back her		
12:15	MM	BB	Baby on her mother back		
12:20	MM	BB	Baby is sleeping on her mother chest		
12:25	BB		Baby sleeping on the mat		
12:30	MM		Mother is washing dishes		
12:35	MM		Mother washing dishes		
12:40	MM		Mother sweeping the surrounding		
12:45	BB,BB2		The baby and her brother are sleeping		
12:50	MM		Mother is packing the dirt's into the dustbin		
12:55	MM		Mother went into the room		
1:00	BB		Baby is still sleeping		
1:05	MM		Mother is packing the clothes she washed earlier to the room		
1:10	MM		Mother fetch water in a bucket and went to the toilet with container carrying sponge and soap		
1:15	MM		The mother came out of the toilet. She is sitting drying her baby with her head tie and gisting with the neighbor		
	BB		The baby is still sleeping		
	MM		The mother goes into the room dressed up and came out		
1:20	BB2		The son wakes up he pissed		
	BB2	MM	Up his body, the mother remove his clothes and bath him, the boy is		

TIME	ACTOR INITIALS	RECIPIENT INITIALS	WHAT HAPPENED	FOOD	Reactivity
			crying		
	MM		The mother puts the clothes in the remaining water add klin and wash them		
1:25	MM	BB2	The mother dressed up the son		
1:30			Baby is still sleeping. The baby wakes up and start crying		
	MM	BB	Mother removes the baby clothes, the baby is easing herself. The mother fetch water to bath her and she ran away and starts crying, she took her and bath her		
1:35			She went into the room and dress her up		
1:40	MM	BB,BB2	The mother brought tuwon dawa with baobab leaves soup for the children and they eat, the baby is feeding herself	Tuwon dawa with baobab soup leaves	
1:45	BB2		They are still eating the food		
	MM	BB,BB2	Mother add soup for them, the baby starts crying, she does not want to allow her brother to eat, the mother said he should stop eating and allow her to finish	Tuwon dawa and dried baobab leaves soup	
1:50	BB		The baby is still eating the food		
1:55	FF		Father comes back with a black leather and give to the mother, she opens it and saw gwade and chin-chin. The gwade is made up groundnut, she gives to the children and they eat	Gwade and chin-chin	
2:00	MM	BB	She adds to the baby		
2:05	MM	BB,BB2	Mother washing the children hands with water in the bowl		
	MM	BB	Mother adds chin-chin to the baby		

TIME	ACTOR INITIALS	RECIPIENT INITIALS	WHAT HAPPENED	FOOD	Reactivity
2:10					
2:15	MM	BB	Mother gave the baby the remaining chi-chin		
2:20	BB	SB	The son is eating tuwon dawa, dried babab leaves soup with the neigbours child	Tuwon dawa with dried baobab leaves soup	
2:25	BB	SB	They are still eating the food	Tuwon dawa with dried baobab leaves soup	
2:30	MM	BB,BB SB	Mother gives the baby, son and neighbours son gwade	Gwade from groundnut	
	BB2	BB	The son is drinking water and he gives to the baby.		
2:35	MM	BB	Baby is making trouble to the mother, the mother back her and she starts smiling. The neighbor says when your mother have another baby you will not do all this		
2:40	MM	BB	Mother still backing the baby		
2:45	MM		Mother keep the baby on the ground		
2:50	BB		The boy is playing		
2:55	BB	BB2	The baby is playing with her brother and she just piss up her legs because she is standing		
	MM	BB	The mother wash her baby		
3:00	BB		Baby playing on her mother body, the mother is lying down on the mat		
3:05	BB		Baby is playing with the neighbor son		

TIME	ACTOR INITIALS	RECIPIENT INITIALS	WHAT HAPPENED	FOOD	Reactivity
3:10	MM	NB	Mother is making bread		
3:15	MM	BB	Baby is crying and she said her mother most remove her clothes for her		
	BB		The baby went out to play with sand		
3:20	BB		Baby is sitting on her mother's lap and the mother is making breads for her neighbour		
3:25	BB,BB2 SB		The baby is playing alone, the son and the neighbor are also playing together		
3:30	MM	NB	Mother still making breads for her neighbor		
3:35	BB		Baby is still playing		
3:40	BB		Baby still playing		
3:45	BB,MM		Baby is playing and the mother is still making breads. The son is lying down on the mat		
3:50			The baby is playing, she ease herself and continue playing		
3:55	BB		Baby still on her mother laps		
4:00	BB	BB2	Baby playing with her brother		
4:05	BB2	BB	Brother is beating his sister and she is crying.		
	MM		The baby run to the mother crying and the mother took her and sit with her on her lap		
4:10	BB2		The son is playing		
4:15	MM	BB,BB2	Mother gives the baby and her brother gwade to eat	Gwade	
4:25	BB2		The son is lying down on a mat		
4:30	MM	BB2	Mother sent the son to bring water for the baby from the pot		

TIME	ACTOR INITIALS	RECIPIENT INITIALS	WHAT HAPPENED	FOOD	Reactivity
	BB		Baby is drinking the water		
4:35	BB		The baby is playing with her mother sitting on her lap		
4:40	FF		Father comes into the house and the mother brought water for him to bath		
	MM		Mother fetch water in a bucket and take to the toilet, took the container, soap and sponge and say to him the water is ready, then the father stood up to the toilet		
4:45	MM	BB,BB2	Mother brought the remaining pap in a bucket and share it to the bay and her brother	Cold millet pap	
4:50	BB		Baby is playing on the flour and the mother starts making breads, the baby starts crying and the mother leave her hair		
4:55	BB		Baby is playing		
5:00	MM		Mother is setting fire		
5:05	MM		Mother put the pot on the fire filled with water		
5:10	BB,BB2		Baby playing with her brother, the brother is refusing		
5:15	BB,BB2 mm		The mother and the children are lying down on a mat, the bay is playing besides her mother		
5:20	BB2		The son starts crying and the mother said he should go to the room and take tuwo and mixed with water.		
	BB2		The son took the bowl take some tuwo dawa from the room come out and mix and he starts drinking it	Tuwon dawa mixed with water	
	BB2		The boy is reducing the water he starts drinking, the baby is		

TIME	ACTOR INITIALS	RECIPIENT INITIALS	WHAT HAPPENED	FOOD	Reactivity
			following him crying		
5:25	MM	BB	Mother fetch water in a cup give the baby and took tuwo dawa and put into the remaining water and mixed, starts giving to the baby	Tuwon dawa mixed with water	
5:30	MM		Mother rinsing the tamarind leaves and putting it into the pot		
	BB2		The son add another tuwo dawa in his bowl and add water and he starts mixing it	Tuwon dawa mixed with water	
5:35	MM	BB	Mother is washing the s body		
	BB		Baby is playing with water		
5:40	MM	BB	Baby is stooling, the mother packed the stool and washed her body		
	BB		Baby is playing		
	BB		Baby is drinking water		
5:45	MM		Mother is sieving flour sorghum		
5:50	MM		Mother is sieving the flour		
	BB		Baby is still playing		
5:55	MM		Mother is draining the boiling water in the pot from the leaves	tamarind leaves	
	MM		Mother fetching the water and backing the pot		
6:00	MM		Mother setting another fire, she put a small pot on the fire and add water then close the pot		
	MM	BB	Mother is playing with the baby		
	BB		The son is outside playing with neighbor children		
6:05	MM		Mother took a small calabash, put		

TIME	ACTOR INITIALS	RECIPIENT INITIALS	WHAT HAPPENED	FOOD	Reactivity
			small amount of sorghum flour, add the boiling water, mix like a ball and put in the boiling water continuously		
	MM		Mother washing dishes and cups		
	MM		Mother took a big silver bowl add the sorghum flour add small amount of cold water and stir, then took the whole boiling tamarind water with some flour ball all into the bowl, its turns to pap (tamarind pap)		
6:15	MM		Mother washing beans in a bowl and adding it on a small pot she put on a fire near her, she add salt and magi	beans	
	MM		Mother took black ashes from a bag and add water into it with another bowl under it. The water is dropping down, she add the water into the beans		
6:20	MM		Mother cutting okra	Okra	
	BB,BB2		The baby is playing with her brither on the ground		

Primary Questions (Complete after you complete data collection):

From what you have seen observing THIS HOUSEHOLD, please answer the following questions in detail:

Question	Write your detailed response here
1. In this household, what would be the best way(s) to give nutritional supplements such as an MNP to young children to ensure compliance?<br><br>Why?	Is through the mother because she takes care of the children, she is in charge of feeding the baby and other things in the house. Secondly, the grandmother , I was told by the neighbor of the grandmother that whenever the mother is going out she drops them there that is with the grandmother and sometimes even if the mother is at home the son (small boy) will come with his sister to the house

2. In this household, who is favored during meal preparation or feeding?<br><br>How is this favoritism expressed?	The bay is favoured during the meal feeding because the mother gives her food first before anyone in the house. When a special food is brought in the house she will have the major shared. If she is crying about and does not want to eat with his brother, the mother will say to the brother to leave it for her. The baby is always protected by her mother.
3. How could we promote the MNP supplement for this family that would attract their attention?<br><br>Who would they listen to?	Through the father because the women in this community really serve their husbands as I saw, not only in this household they respect and obey their husband's instruction. Therefore, I think the best way to promote MNP for this family is through the husband
4. In this household, what would be the best ways to promote the MNP but limit how much it would be shared?<br><br>Why?	Through proper sensitization of the product to the mothers because she is the person feeding the baby and other children in the house. So she can make sure that baby eats/takes th MNP alone without sharing it with others.<br>-enough information about the MNP<br>-how to administer the MNP<br>-rules and regulations of MNP
5. What other issues and concerns did you see with this family that are important for us to consider with regard to introduction of an MNP?<br><br>Why?	Is sharing the MNP with neighbor children that do not have the MNP because they came to the mother to give them some food to give their children and some say that we should please give this MNP to their children although they are not among the selected ages so that they will be healthy<br>Inadequate food is also a concern in this family, sorghum is the major food eaten in this house
6. Based on your experience observing this child today, do you believe reactivity negatively impacted your ability to see the food-related behaviors of the child?	Based on my experienced I don't think reactivity negatively impacted on my ability to see the food related behaviours of the child because the neighbor says the moment the mother have another baby, she will not have all this care, so I learnt that when you have a baby in the house before the mother have another baby, you will have everything (caring and feeding) is special. The

Please explain your answer in detail.	moment she has another baby, the baby takes over. Therefore, she is being favoured because she is the baby of the house
